# FKBP51 increases the tumour-promoter potential of TGF-beta

**DOI:** 10.1186/2001-1326-3-1

**Published:** 2014-01-27

**Authors:** Simona Romano, Anna D’Angelillo, Paolo D’Arrigo, Stefania Staibano, Adelaide Greco, Arturo Brunetti, Massimiliano Scalvenzi, Rita Bisogni, Iris Scala, Maria Fiammetta Romano

**Affiliations:** 1 Department of Molecular Medicine and Medical Biotechnologies, Federico II University, via Pansini, Naples 5. 80131, Italy; 2 Department of Advanced Biomedical Sciences, Federico II University, Naples, Italy; 3 CEINGE Biotecnologie Avanzate, Naples, Italy; 4 Department of Clinical Medicine and Surgery, Dermatology Section, University Federico II of Naples, Naples, Italy; 5 Department of Medical and Translational Sciences, Pediatrics Section, University Federico II of Naples, Naples, Italy

**Keywords:** Melanoma, TGF-β, FKBP51, Metastasis

## Abstract

**Background:**

FKBP51 (FKBP5 Official Symbol) is a large molecular weight component of the family of FK506 binding proteins (FKBP). In recent years, research studies from our laboratory highlighted functions for FKBP51 in the control of apoptosis and melanoma progression. FKBP51 expression correlated with the invasiveness and aggressiveness of melanoma. Since a role for TGF-β in the enhanced tumorigenic potential of melanoma cells is widely described, we hypothesized a cooperative effect between FKBP51 and TGF-β in melanoma progression.

**Methods:**

SAN and A375 melanoma cell lines were utilized for this study. Balb/c IL2γ NOD SCID served to assess the ability to colonize organs and metastasize of different cell lines, which was evaluated by in vivo imaging. Realtime PCR and western blot served for measurement of mRNA and protein expression, respectively.

**Results:**

By comparing the metastatic potential of two melanoma cell lines, namely A375 and SAN, we confirmed that an increased capability to colonize murine organs was associated with increased levels of FKBP51. A375 melanoma cell line expressed FKBP51 mRNA levels 30-fold higher in comparison to the SAN mRNA level and appeared more aggressive than SAN melanoma cell line in an experimental metastasis model. In addition, A375 expressed, more abundantly than SAN, the TGF-β and the pro angiogenic TGF-β receptor type III (TβRIII) factors. FKBP51 silencing produced a reduction of TGF-β and TβRIII gene expression in A375 cell line, in accordance with previous studies. We found that the inducing effect of TGF-β on Sparc and Vimentin expression was impaired in condition of FKBP51 depletion, suggesting that FKBP51 is an important cofactor in the TGF-β signal. Such a hypothesis was supported by co immunoprecipitation assays, showing that FKBP51 interacted with either Smad2,3 and p300. In normal melanocytes, FKBP51 potentiated the effect of TGF-β on N-cadherin expression and conferred a mesenchymal-like morphology to such round-shaped cells.

**Conclusions:**

Overall, our findings show that FKBP51 enhances some pro oncogenic functions of TGF-β, suggesting that FKBP51-overexpression may help melanoma to take advantage of the tumor promoting activities of the cytokine.

## Background

FK506 binding protein 51 (FKBP51) [1] is an immunophilin physiologically expressed in lymphocytes and several other tissues [2]. FKBP51 structure includes C-terminal TPR domains, responsible for protein protein interactions with heat shock proteins HSP90 and HSP70, and N-terminal domains with peptidyl-prolyl isomerase activity [1]. Due to its multifunctional domains, FKBP51 regulates several biological processes in the cell, through protein-protein interaction [1]. Very recently, we found an aberrant expression of this protein in melanoma [3,4]. We demonstrated that FKBP51 promotes activation of epithelial-to-mesenchymal transition (EMT) genes and improves melanoma cell migration and invasion [4]. Consistent with this finding, FKBP51-targeting prevented melanoma colonization of liver and lungs in a mouse model of experimental metastasis [4]. The pleiotropic cytokine TGF-β plays a relevant role in EMT [5]. Notably, TGF-β acts as an early tumor suppressor, but functions later as a tumour promoter and a pro-metastatic agent [6]. In normal melanocytes, TGF-β acts as a potent inhibitor of proliferation and differentiation [7,8]; in advanced melanoma, TGF-β favors cell proliferation and dissemination, peri-tumoral angiogenesis, EMT and tumor escape from immune surveillance [9-11]. The mechanism underlying evasion from a cytostatic response to TGF-β in tumor cells remains somewhat elusive. Our previous results showed that FKBP51 positively regulates the expression of TGF-β, in melanoma [4]. We hypothesized a role for FKBP51 in potentiating the tumour promoting activities of TGF-β, in melanoma.

## Methods

### Cell culture and transfection and reagents

The melanoma cell lines SAN and A375 were cultured as described [3]. For siRNA transfection, 24 hours before transfection, cells were seeded into six-well plates at a concentration of 2×10^5^ cells/ml to obtain 30–60% confluence at the time of transfection. Then, cells were transfected with specific short-interfering oligoribonucleotide (siRNA) or with a non silencing oligoribonucleotide (NS RNA) as control, at a final concentration of 50 nM using Metafectene according to the manufacturers’ recommendations. NS RNA (AllStars neg control siRNA) and siRNA for FKBP51 (5′-ACCUAAUGCUGAGCUUAUA-3) were purchased from Qiagen (Germantown, Philadelphia, USA). ShRNA transfection was performed using the Expression ArrestTM shRNA system (Open Biosystem, AL, USA). Expression ArrestTM shRNAs are cloned into the incompetent replication pSHAG-MAGIC2 (pSM2) retroviral vector. This vector has a Murine Stem Cell Virus (MSCV) backbone combined with packaging extract for mammalian cell infection, a PGK- Puro selection for transfection stability in mammalian cells and a chloramphenicol/kanamycin bacterial selection marker. The stable transfectants were obtained after a 1 month selection of positive clones. The selection was performed by adding puromycin (Sigma Aldrich, Saint Louis, Missouri, USA) to cell culture medium every 48 hours. For a first stronger selection puromycin was used at a dose of 800 ng/ml; after a week it was used a dose of 200 ng/ml to complete and maintain the selection. To create FKBP51 over expressing SAN melanoma cells, a p3XFLAG-CMV™-14 expression vector (Sigma Aldrich, S. Louis, Missouri, USA) carrying the FKBP51 gene was transfected using Metafectene (Biontex, Munich, Germany), according to manufacturer’s recommendations. A void p3×Flag-CMV vector was also transfected to generate control cells. To generate stable populations, cells were selected using 500 ug/ml G418 (GIBCO, Invitrogen, Carlsbad, CA) at 24 h post-transfection and grown until colony formation. TGF-β (Sigma Aldrich) was used at the dose of 10 ng/ml.

### Animal studies

After the approval of the local institutional animal research committee, animal studies were performed following detailed internal regulations devised according to the U.S. Public Health Service Policy on Humane Care and Use of Laboratory Animals, available from the Office of Laboratory Animal Welfare, National Institutes of Health, Department of Health and Human Services, RKLI, Suite 360, MSC 7982, 6705 Rockledge Drive, Bethesda, MD 20892–7982 and the United Kingdom Coordinating Committee on Cancer Prevention Research's Guidelines for the Welfare of Animals in Experimental Neoplasia (published online 25 May 2010). Melanoma cells (1.5×10^6^ SAN or A375 in 100 μl PBS) were injected systemically into the lateral tail vein of 4- to 6-week-old Balb/c IL2γ NOD SCID (null) mice (Charles River Laboratory, Wilmington, MA). After 3 weeks, imaging was performed using a dedicated animal PET/CT scanner (eXplore Vista, GE Healthcare). A dose of 8.3 mCi/kg (307.1 MBq/kg) of ^[18]^F-FDG was administrated in a bolus in a total volume of 100 μl. Animals were maintained at a temperature of 23°C during the biodistribution of ^[18]^F-FDG. After 45 minutes, mice were anesthetized with ketamine 50 mg/kg and xylazine 40 mg/kg and symmetrically positioned on a warm bed with micropore tape. Then, a 20-min static PET (two bed position with a 4.8-cm axial field-of-view; energy window 250–700 keV) scan was performed. PET images were processed using a 2D-OSEM iterative algorithm (voxel size of 0.3875 × 0.3875 × 0.7750 mm^3^) including random scatter correction, dead time, decay, and attenuation correction using CT data (eXplore Vista Software).

### Western blot and immunoprecipitation

Whole cell lysates were homogenized in modified RIPA [12] buffer as described and assayed in Western blot as described [3]. Primary antibodies against FKBP51 (F-13; goat polyclonal; Santa Cruz Biotechnology, CA, USA); Smad 2/3 (H465, rabbit polyclonal; Santa Cruz Biotechnology); SPARC (H-90, rabbit polyclonal; Santa Cruz Biotechnology); N-Cadherin (5D5, mouse monoclonal; Abcam, Cambridge, UK); G3PDH (D16H11, rabbit monoclonal; Cell Signaling, Danvers, USA); were used diluted. 1:500. For immunoprecipitation (IP), 500 ug of total lysate was precleared for 1 hour. Three μg anti-KAT3B/p300 (Novus Biologicals, Littleton, CO, USA) or anti-FKBP51 (H100, rabbit polyclonal, Santa Cruz Biotechnology), was added to total lysate, kept in rotation, at 4°C over night. After, 25 uL protein A Agarose (Santa Cruz Biotechnology) was added to the mixture and precipitation took place for 4 h, with rotation at 4°C. Samples were then washed in RIPA and separated by 10% SDS-PAGE. Anti-KAT3B/p300 (Novus Biologicals), anti-FKBP51 (mouse polyclonal; Abnova, Taipei, Taiwan) were used for detection of pulled down proteins.

### Real-time PCR

Total RNA was isolated from cells using Trizol (Invitrogen, Carlsbad, CA, USA) according to the manufacturer’s instructions. One microgram of each RNA was used for cDNA synthesis with Moloney Murine Leukemia Virus Reverse Transcriptase (M-MLV RT, Invitrogen, Carlsbad, CA, USA). Gene expression was quantified by Real-time PCR using iQ™SYBR®Green Supermix (Biorad, CA, USA) and specific Real-time validated QuantiTect primers for FKBP51 (QT00056714: NM_001145775 800 e 900 bp; NM_001145776 650 and 750 bp; NM_001145777 650 and 750 bp; NM_004117 600 and 700 bp); TGF-β (QT00000728: NM_000660 1200 and 1300 bp), TβRIII (QT00013335: NM_000118 600 and 750 bp; NM_001114753 600 and 750 bp), N-cadherin (QT00063196: NM_001792 2800 and 2900 bp) (Qiagen, Germantown, Philadelphia, USA), cyclin B (QT00006615: NM_031966 1250 and 1350 bp), vimentin (QT00095795: NM_003380 900 e 1000 bp), slug (QT00044128: NM_003068200 e 350 bp). Relative quantitation of the transcript was performed using co-amplified ribosomal 18S as an internal control for normalization. Ribosomal 18S primers Fw 5′-CGATGCGGCGGCGTTATTC-3′ and 18S Rev 5′-TCTGTCAATCCTGTCCGTGTCC-3′.

### Flow cytometry

Vimentin expression was measured in flow cytometry. Briefly, after centrifugation for 5 min at 400× g, 1×10^5^ cells were fixed with 2% paraformaldehyde in Tris Buffered Saline solution (TBS) for 20 min and permeabilized with 0.1%TRITON-X-100 and 0.1% Sodium Citrate in TBS for 3 min in ice. Cells were, then, incubated with the mouse monoclonal antibody anti-Vimentin, IgG1 clone-V9; 1:100 (Novocastra™, Milan, Italy) or a control IgG, for 30 min at 4°C. After incubation, cells were washed and stained with a secondary FITC-conjugated anti-mouse antibody and analyzed in flow cytometry.

### Primary melanocyte cultures

Human melanocytes were isolated from an acquired melanocytic naevus, surgically excised, which was obtained after informed consent of the subject, and grown in Melanocyte Medium BulletKitTM - 500 ml CloneticsTM MGMTM-4 BulletKitTM (CC-3249) containing the following growth supplements: CaCl2, 1.0 ml; BPE, 2.0 ml; rhFGF-B, 1.0 ml; rh-Insulin, 1.0 ml; Hydrocortisone, 0.5 ml; PMA, 0.5 ml; GA-1000, 0.5 ml; FBS, 2.5 ml (Gibco, Grand Islands, NY, USA). Briefly, the naevus was placed in sterile phosphate buffered saline (PBS) solution. Subcutaneous fat and deep dermis were excised from the sample, and the remaining tissue was cut into smaller pieces, followed by trypsinization (0.25% trypsin, Gibco) at 37°C for 30 min. Trypsin activity was neutralized with FBS. Each piece was placed under a sterile glass in order to press the tissue and favor cells leaking. Cells isolated from melanocytic naevus started to growth out of the tissue pieces after a couple of weeks. Thenafter, tissues were eliminated and contaminating fibroblasts were selectively killed by treating the cultures with 100 μg/ml G418 for 3–4 days. Melanocytes were subsequently isolated from keratinocytes by gentle trypsinization and passed at a ratio of 1:3 once every 7–14 days. After 1 month, melanocytes were transfected with a p3XFLAG-CMV™-14 expression vector (Sigma Aldrich, S. Louis, Missouri, USA) carrying the FKBP51 gene, or a void vector as control, Metafectene (Biontex, Munich, Germany). After a 3day culture, 25 ng/ml TGF-β for further 3 days; then a picture from different melanocyte cultures was captured and cells were harvested and processed for Real time-PCR assay.

## Results and discussion

### The enhanced tumorigenic potential of melanoma cells is accompanied by increased levels of FKBP51 and TGF-β

**Figure 1:**
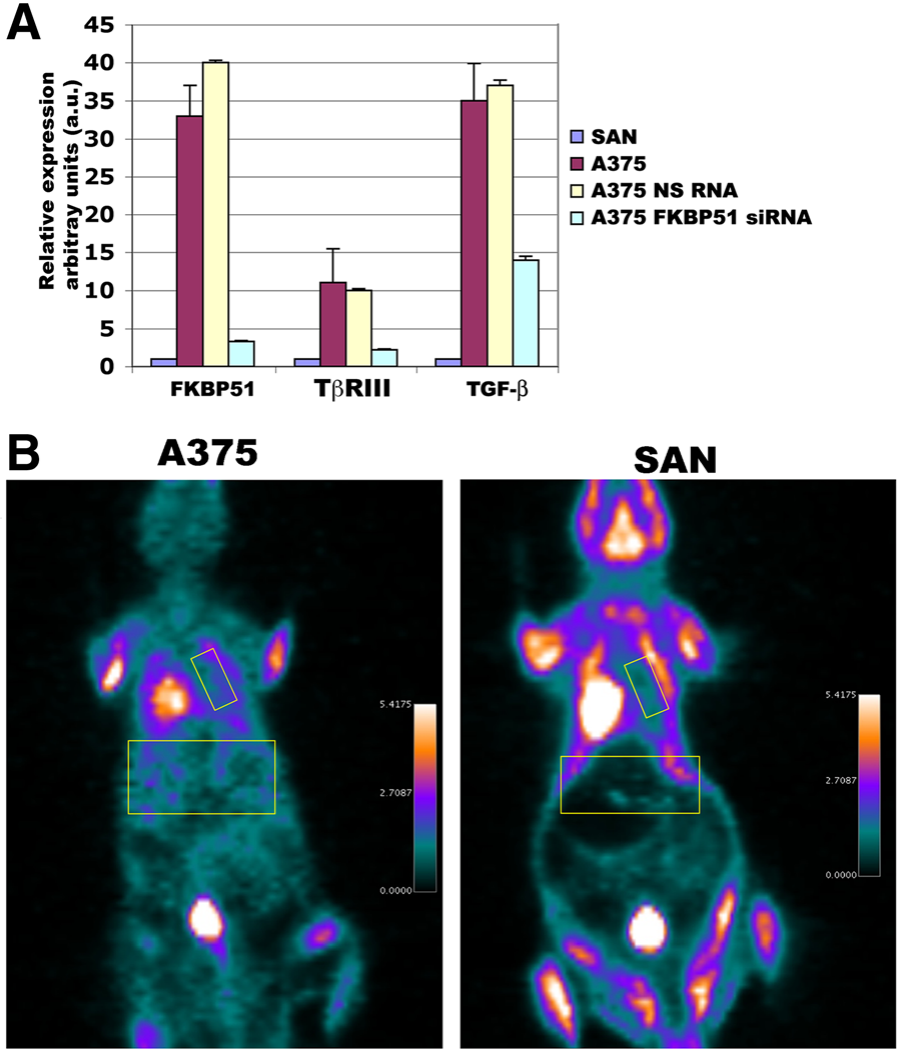
**High FKBP51 expression is accompanied by increased metastatic potential. A**, Real-time PCR measurement of FKBP51, TβRIII, and TGF-β mRNAs in A375 melanoma cells, which were silenced (FKBP51 siRNA) or not (NS RNA) for FKBP51. The relative change of expression in A375 samples was estimated relative to SAN samples (expression = 1). Reduced mRNA levels in FKBP51-silenced A375 confirmed that the immunophilin regulated the expression of TβRIII, and TGF-β in melanoma. **B**, FDG PET coronal views of mouse models of melanoma metastasis, 21 days after iv injection of two different cell lines: A375 (left) and SAN (right). FDG PET imaging show a diffuse and more prominent FDG uptake (mostly at lung and liver level, yellow squares) in the left mouse, suggesting that A375 have enhanced tumorigenic potential compared with SAN.

We have previously shown that SAN melanoma cells injected into the tail vein of immunosuppressed mouse produced significant liver and lung colonization within 4 weeks, and such invasive potential was strictly dependent on the expression of FKBP51 [4]. In the present study, we compared the expression of FKBP51 in two different melanoma cell lines, namely A375 and SAN, and the capability of these cell lines to form in vivo metastasis. A375 melanoma cell line expressed FKBP51 mRNA levels 30-fold higher in comparison with the SAN cell line (Figure 1A). In addition, A375 expressed, more abundantly than SAN, the TGF-β and the pro angiogenic TGF-β receptor type III (TβRIII) factors [4]. FKBP51 silencing produced a reduction of TGF-β and TβRIII gene expression in A375 cell line (Figure 1A), which is in accordance with the upregulation of these genes by FKBP51 [4]. Injection of SAN and A375 cells into the tail vein of immunosuppressed mice showed an increased capability of A375 to colonize murine organs, in comparison with SAN. In fact, FDG PET imaging performed after 21 days after iv, showed a diffuse and more prominent FDG uptake, mostly at lung and liver level (yellow squares) in mouse injected with A375 (Figure 1B). These results confirm previous findings of an association of the aggressive behaviour and high FKBP51 [4] and TGF-β [7] levels, in melanoma.

### FKBP51 positively regulates the TGF-β signal in melanoma

**Figure 2:**
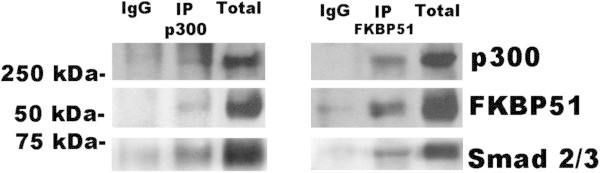
**FKBP51 interacts with the general transcriptional co-activator p300 and the TGF-β transcription factor Smad2/3.** Left; FKBP51 co immunoprecipitates with p300. Right; p300 co immunoprecipitates with FKBP51. Total cell lysates were prepared by SAN melanoma cells transfected with FKBP51/Flag. Cell lysates were immunoprecipitated with anti-Kat3B/p300 (IP p300) or anti-Flag (IP FKBP51). Immunoprecipitated and total lysates were then subjected to Western blot with anti-FKBP51, anti-p300 or anti-Smad 2/3. Smad 2/3 co immunoprecipitate with either p300 (left) and FKBP51 (right).

The induction of components and receptors of the TGF-β family, can occur through the action of TGF-β factors themselves [13]; we hypothesized a positive regulation of the TGF-β signal by FKBP51. Consistent with this hypothesis, type I TGF-β receptor (TβRI), a direct transcriptional target of TGF-β, [13]- was found increased as a consequence of FKBP51-overexpression (Additional file 1: Figure S1). As known, TβRI, or activin receptor-like kinase, phosphorylates, hence activates, the transcriptional factors Smad2 and Smad3 enabling them to translocate into the nucleus [14]. The cellular context is central in determining which genes will respond to an activated Smad complex as it arrives in the nucleus [14]. Several proteins are described that facilitate Smad ability to recruit coactivators [14]. We have previously shown that FKBP51 interacts with p300 [4], one of the major coactivators of Smad 2,3 [15]. To address whether FKBP51 can modulate the TGF-β signal, we, performed co immunoprecipitation assays to investigate whether Smad 2/3 participated to the FKBP51/p300 complex. As shown in Figure 2, we confirmed that FKBP51 co immunoprecipitated with p300, and, conversely, p300 co immunoprecipitated with FKBP51, in line with previous study [4], and provided evidence that this interaction involved also Smad 2/3 that co immunoprecipitated with either p300 and FKBP51 (Figure 2).

**Figure 3:**
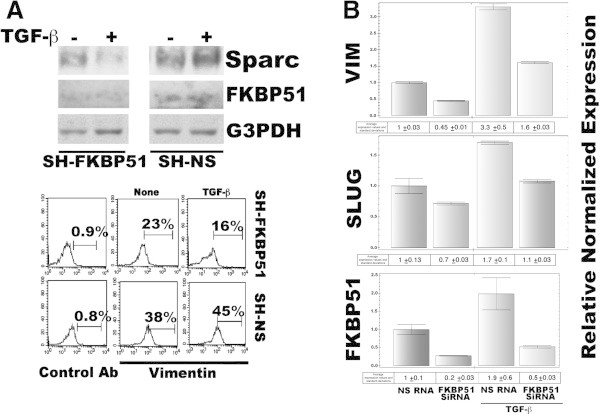
**FKBP51 enhances expression of pro oncogenic factors in SAN melanoma cells stimulated with TGF-β. A**, upper Western blot assay of Sparc levels in cell lysates obtained from the melanoma cell line SAN transfected with a specific FKBP51 shRNA (SH-FKBP51), or a non-silencing (SH-NS) shRNA as control, in the absence or the presence of 10 ng/ml TGF-β. After a 18 h culture, cell was harvested for lysates preparation. **A**, lower Flow cytometric histograms of Vimentin expression in SH-FKBP51 or SH-NS, in the absence or the presence of 10 ng/ml TGF-β. Cell was harvested after a 18 h culture. Vimentin was measured by indirect immunofluorescence, in fixed and permeabilized cells. **B** Effect of FKBP51 siRNA on VIM and SLUG expression levels*.* Normalized expression rates (mean±s. d.) of VIM (upper), SLUG (intermediate) and FKBP51 (lower) mRNA levels. NS RNA-treated sample expression=1 (N=2). Melanoma cell line SAN was transfected with a specific FKBP51 siRNA, or a non-silencing (NS) RNA as control. After 24 h from transfection 10 ng/ml TGF-β was added to the cultures. Cell was harvested after further 18 h and total RNA was extracted.

**Figure 4:**
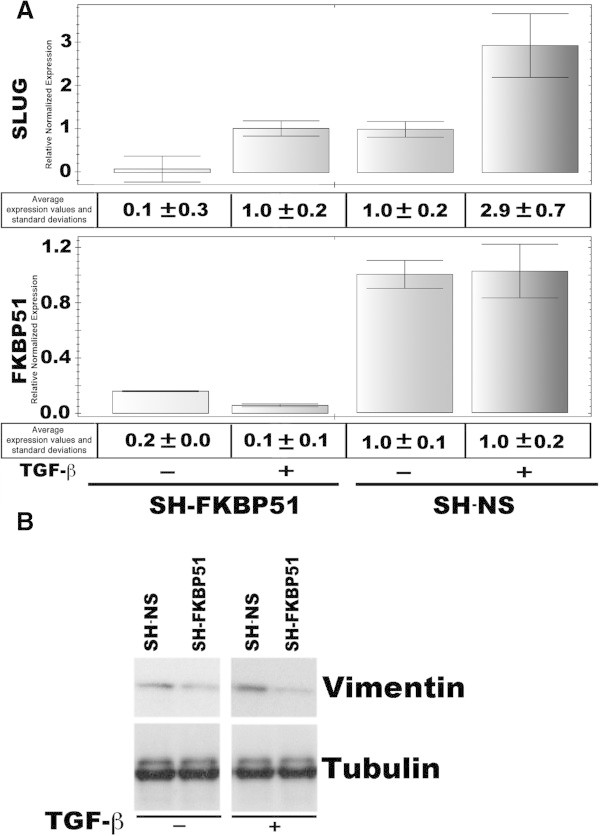
**FKBP51 enhances expression of pro oncogenic factors in A375 melanoma cells stimulated with TGF-β. A** Normalized expression rates (mean±s. d.) of SLUG (upper), and FKBP51 (lower) mRNA levels. SH-NS sample expression=1 (N=2). RNA was extracted from the melanoma cell line A375 stably transfected with a specific FKBP51 shRNA (SH-FKBP51), or a non-silencing (SH-NS) shRNA as control, in the absence or the presence of 10 ng/ml TGF-β. After a 18 h culture, cell was harvested. **B** Western blot assay of vimentin expression in whole cell lysates prepared from SH-NS and SH-FKBP51 cells cultured in the absence or the presence of 10 ng/ml TGF-β for 18 h. TGF-β stimulated an increase in vimentin level in SH-NS but not SH-FKBP51 cells. Reduced basal levels of vimentin were observed in SH-FKBP51 cells.

**Figure 5:**
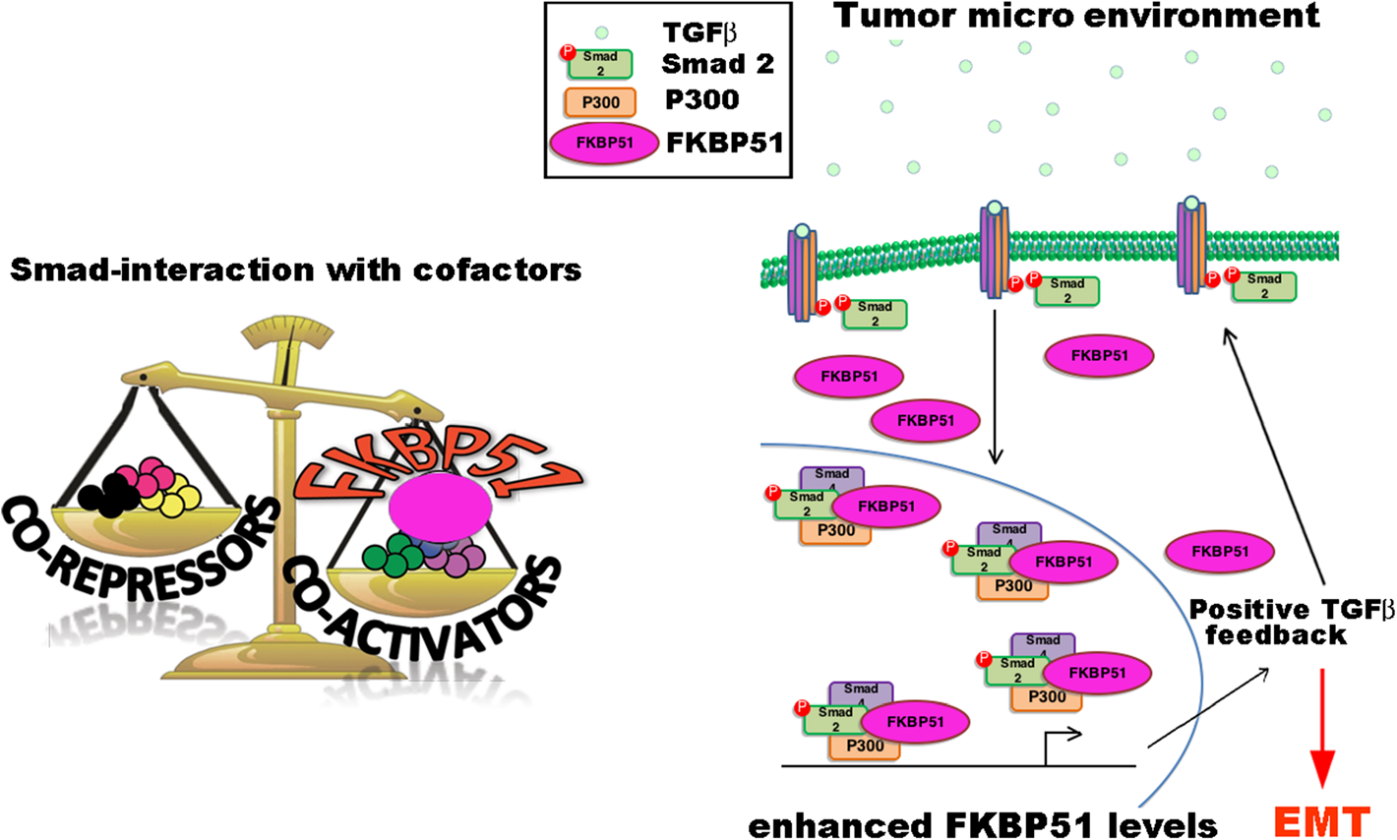
**Mechanism proposed for FKBP51 enhancement of TGF-β pro-oncogenic signal.** Left, FKBP51 facilitates Smad recruitment to coactivators. Right, FKBP51 takes part to the transcriptional complex formed by P300 and Smad 2,3 Increase in FKBP51, as it occurs in melanoma, generates an auto regulatory loop of TGF-β signaling, which in turn promotes tumour progression.

To address whether FKBP51 may promote a pro-oncogenic signal, we, evaluated the TGF-β-induced expression of two typical markers of melanoma aggressiveness, under TGF-β-transcriptional control, namely Sparc [16], and Vimentin [17], in melanoma cells stably silenced for FKBP51 with short hairpin RNA (SH-FKBP51) or transfected with a non silencing short hairpin as control (SH-NS). As known, Sparc is the secreted protein acidic and rich in cysteine, that is regulated in tissues undergoing remodeling, during normal development, tissue repair, and in cancer [18]. Increased expression of Sparc is associated with aggressive tumor phenotype in melanomas and gliomas [19]. Vimentin is a major constituent of the intermediate filament family of proteins, is ubiquitously expressed in normal mesenchymal cells. Vimentin's overexpression in cancer correlates well with accelerated tumor growth, invasion, and poor prognosis [20]. Levels of Sparc and Vimentin were measured in western blot (Figure 3A, upper) and flow cytometry (Figure 3A, lower), respectively. Such levels appeared higher in control cells, compared to FKBP51 knocked down cells. Our data showed TGF-β increased expression of Sparc and Vimentin in SH-NS cells, but not SH-FKBP51 cells. These results suggest a role for FKBP51 in promoting some pro-oncogenic activity of TGF-β. Expression and activation of the EMT regulatory factor SLUG is driven by SPARC [21]. We used SAN melanoma cells that were in transient transfected with FKBP51 siRNA or a non silencing (NS) RNA as control, and measured by QPCR levels of SLUG and VIM mRNA, in the absence or the presence of TGF-β. A representative result of two independent experiments is shown in Figure 3B. Reduced levels of SLUG and VIM were measured in FKBP51-downmodulated melanoma, either in the absence and the presence of TGF- β, in comparison with levels measured in non silenced cells. Similar results have been obtained with A375 melanoma cell line (Figure 4). Figure 5 represents schematically the proposed mechanism for FKBP51 regulation of the TGF-β signaling. FKBP51 can guide the choice of Smad cofactor on p300 (Figure 5, left). In melanoma, the increased expression of FKBP51 creates a positive feed-back of the TGF-β signal which in turn promotes tumoral progression (Figure 5, right).

### FKBP51 upregulates EMT features in TGF-β-cultured normal skin melanocytes

**Figure 6:**
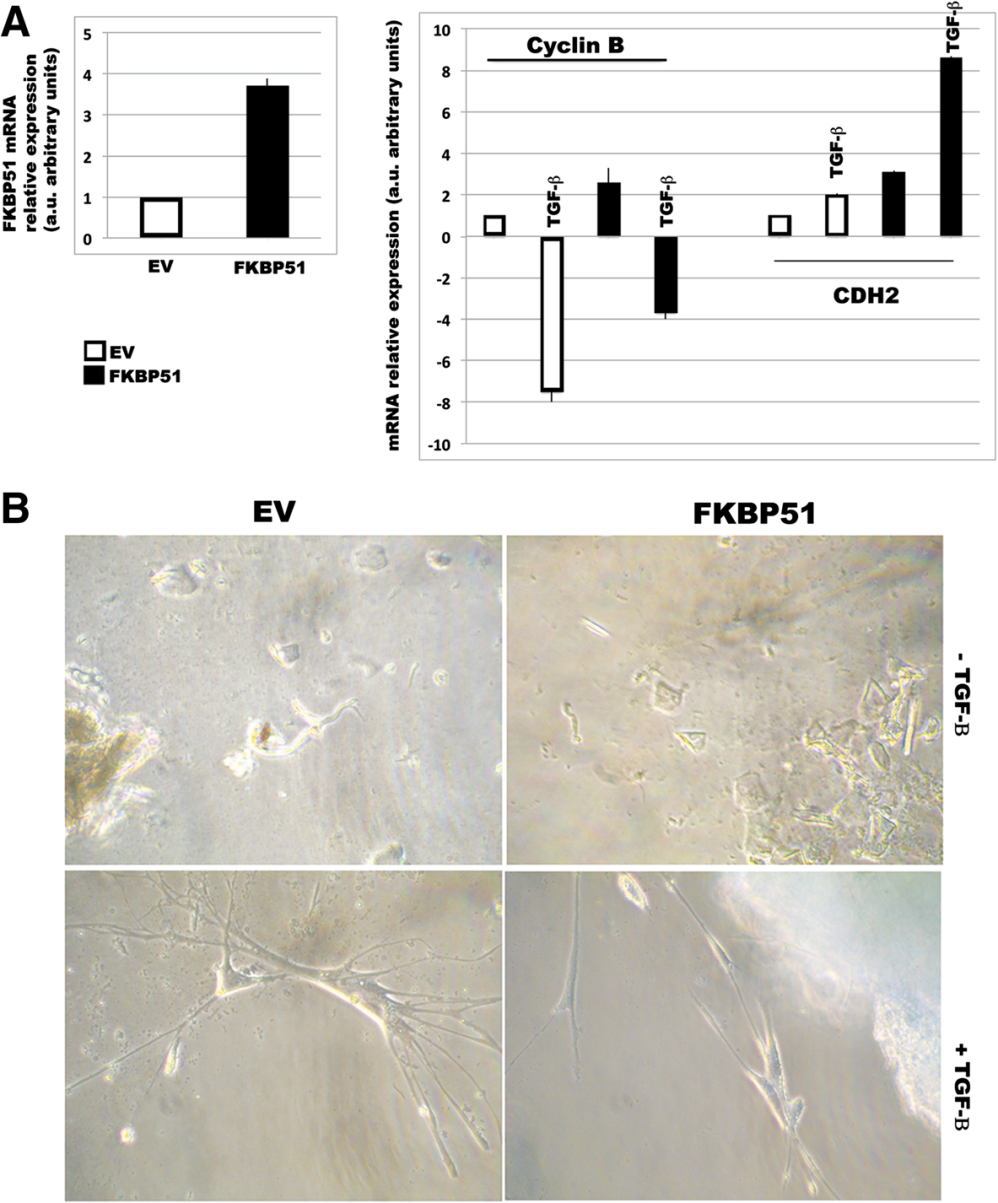
**FKBP51 modulates the TGF-β signal in normal melanocytes. A**, left Real-time PCR measurement of FKBP51, mRNAs in melanocytes, which were transfected with FKBP51 plasmid or the empty vector (EV) as control. The enhancement of FKBP51 transcript validated the efficacy of transfection. **A**, right Real-time PCR measurement of Cyclin B and CDH2 mRNAs in melanocytes, FKBP51- or EV-transfected, cultured in the absence or the presence of 10 ng/ml TGF-β for 3 days. The relative change of expression in different samples was estimated relative to EV sample (expression = 1). **B**, Phase contrast microscopy of melanocytes FKBP51- or EV-transfected, cultured in the absence or the presence of 10 ng/ml TGF-β for 3 days. Upper; EV- or FKBP51-melanocytes presented rounded/polygonal morphology. Lower left; EV- melanocytes cultured with TGF-β presented slender spindle shape with multiple dendritic extensions. Lower right; FKBP51-melanocytes cultured with TGF-β displayed elongated shape and bipolar spindle morphology.

We then used primary melanocytes isolated from epidermis that express no or low levels of FKBP51 [3], to investigate the effect of exogenous FKBP51 on TGF-β response. Melanocytes were transfected with FKBP51 and, after 3 days, TGF-β was added to the cultures. After further 3 days, cell was harvested, and RNA was extracted. Figure 5A shows that melanocytes transfected with FKBP51 plasmid contained FKBP51 transcript level increased by more than 3-fold, in comparison with melanocytes transfected with empty vector. TGF-β produced a 7-fold decrease of cyclin-B expression, in accordance with the notion that this cytokine exerts anti-proliferative effect on normal melanocytes [7]. In FKBP51 overexpressing melanocytes, cyclin B remained suppressed in the presence of the cytokine, although to a lesser extent, in comparison with EV-melanocytes. Differently, TGF-β induced a 2-fold increase of N cadherin levels in melanocytes transfected with the empty vector, and an 8-fold increase in condition of FKBP51 overexpression. Morphological examination of melanocyte cultures in phase contrast microscopy showed rounded/polygonal melanocytes (Figure 6, upper) in unstimulated cultures. In TGF-β cultures, melanocytes transfected with empty vector presented slender spindle shape with multiple dendritic extensions (Figure 6, lower left). FKBP51-transfected melanocytes displayed elongated shape and bipolar spindle, i.e. mesenchymal morphology, which is in line with increased N cadherin levels (Figure 6, lower right). These results suggested that FKBP51, in TGF-β-stimulated normal melanocytes can promote some EMT traits, that are related to a de differentiation process [7]. Such a mechanism may assume a pathogenetic significance during melanocyte transformation.

## Conclusions

In melanoma, several models of resistance to TGF-β growth suppressor signals have been suggested, also in view of the dysregulation of fundamental cellular effectors and signaling pathways [9,22]. We propose FKBP51 may represent an element, within melanoma cell context, that allows the tumour to take advantage of tumour-promoting activities of the TGF-β. Our findings suggested that FKBP51 increases melanoma sensitivity to TGF-β, and, particularly, the tumour promoting activities of the cytokine, possibly mediated by a mechanism involving recruitment of Smad to p300 coactivator. The differential expression of FKBP51 in normal melanocytes, in which the immunophilin is hardly detectable [3], and melanoma, in which the protein is overexpressed, might in part account for differential functions exerted by TGF-β in normal and malignant melanocytes. In addition, the concept that FKBP51 expression increases with melanoma progression, is also in accordance with the notion that TGF-β acts as an early tumor suppressor and late tumour promoter [5,6,14].

## Abbreviations

FKBP51: FK506 binding protein 51; TGF-β: Transforming growth factor-β; TβRIII: TGF-β receptor type III; TβRI: TGF-β receptor type I; EMT: Epithelial to mesenchymal transition; siRNA: Short-interfering RNA; NS RNA: Non silencing RNA; SHRNA: Short-hairpin RNA.

## Competing interests

The authors declare that they have no competing interests.

## Authors’ contributions

S.R. and M.F.R. planned the project. S.R., A.D., P.D., R.B., and IS. carried out experimental work. A.G. and A.B. performed animal studies and imaging. S.S. and M.M. provided clinical information, human tissues and microscopy studies, M.F.R. wrote the paper. All authors discussed the results and commented on the manuscript. All authors read and approve the final manuscript.

## Supplementary Material

Additional file 1: Figure S1 *Increased T*β*RI expression in FKBP51 overexpressing melanoma.* Left, normalized expression of TβRI and FKBP51 mRNA in WT, EV-, or FKBP51-stably transfected SAN melanoma cells. WT sample expression=1. (N=3). Right, normalized expression of TβRI and FKBP51 mRNA in SAN melanoma cells transfected with FKBP51 siRNA or a non silencing RNA as control. NS sample expression=1. (N=3). Click here for file
