# Supplementary figures and images for: FKBP51 increases the tumour-promoter potential of TGF-beta
Source: Clin Transl Med. 2014 Jan 27;3:1. doi: 10.1186/2001-1326-3-1 (PMC3906759; doi:10.1186/2001-1326-3-1)

## Slide 1
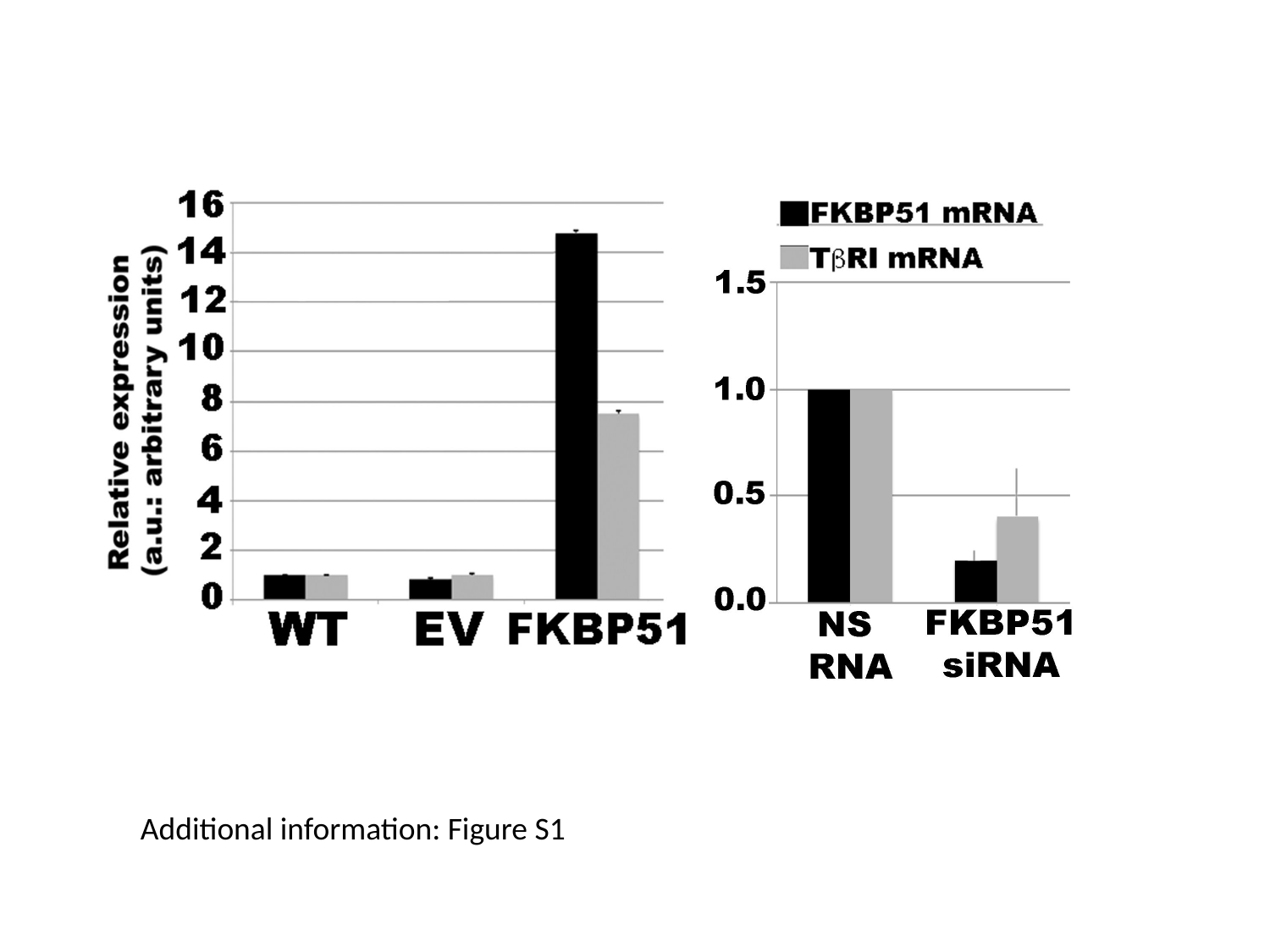

Additional information: Figure S1

Supplement: Additional file 1: Figure S1 — Increased TβRI expression in FKBP51 overexpressing melanoma. Left, normalized expression of TβRI and FKBP51 mRNA in WT, EV-, or FKBP51-stably transfected SAN melanoma cells. WT sample expression=1. (N=3). Right, normalized expression of TβRI and FKBP51 mRNA in SAN melanoma cells transfected with FKBP51 siRNA or a non silencing RNA as control. NS sample expression=1. (N=3). [file 2001-1326-3-1-S1.pptx]
